# Antiviral Sesquiterpene Lactones from *Laurus nobilis* L.: Inhibition of Hepatitis C Virus and Dengue Virus Replication

**DOI:** 10.3390/v18101056

**Published:** 2026-09-23

**Authors:** Yhiya Amen, Marwa Elsbaey, George Mpekoulis, Evangelia Christou, Helena Tsimpili, Niki Vassilaki, Kuniyoshi Shimizu, Grigoris Zoidis

**Affiliations:** 1 Department of Pharmacognosy, Faculty of Pharmacy, Mansoura University, Mansoura 35516, Egypt; yhiaamen@mans.edu.eg (Y.A.); marwaelsebay1611@mans.edu.eg (M.E.); 2 Department of Agro-Environmental Sciences, Graduate School of Bioresource and Bioenvironmental Sciences, Kyushu University, Fukuoka 819-0395, Japan; 3 Molecular Virology, Hellenic Pasteur Institute, 11521 Athens, Greece; g.mpekoulis@gmail.com (G.M.); lillo0ochristou@gmail.com (E.C.); nikiv@pasteur.gr (N.V.); 4 Division of Pharmaceutical Chemistry, Department of Pharmacy, School of Health Sciences, National and Kapodistrian University of Athens, Panepistimiopolis-Zografou, 15771 Athens, Greece; etsimpili@pharm.uoa.gr; 5 Institute of Asian and Oceanic Studies, Kyushu University, Fukuoka 819-0395, Japan

**Keywords:** *Laurus nobilis*, dehydrocostus lactone, costunolide, sesquiterpene lactones, hepatitis C virus, dengue virus, antiviral activity, natural products

## Abstract

Natural products remain indispensable scaffolds in antiviral drug discovery, particularly against high-burden viral pathogens. In this study, two major sesquiterpene lactones, dehydrocostus lactone (**1**) and costunolide (**2**), were isolated from *Laurus nobilis* L. leaves and evaluated for their antiviral potential against hepatitis C virus (HCV) and dengue virus (DENV). The structures of both compounds were confirmed by HRMS and 1D NMR spectroscopic analyses. In subgenomic replicon assays, the molecules inhibited HCV replication in the low-micromolar range (${\mathrm{EC}}_{50}=2.34\;\mu M$ for **1** and 2.06 μM for **2**) with favorable selectivity indices. In full-length HCV reporter systems, costunolide (**2**) displayed enhanced anti-HCV potency, reduced viral RNA and NS5A protein levels, and enhanced the suppressive effect of the clinically approved NS5A inhibitor, daclatasvir. Furthermore, costunolide (**2**) exhibited cross-flaviviridae activity, suppressing DENV replication (${\mathrm{EC}}_{50}=5.89\;\mu M$ in replicon cells; ${\mathrm{EC}}_{50}=1.27\;\mu M$ post-infection in infectious virus assays), and demonstrated cooperative suppression of viral propagation along with reduced glutathione. Mechanistically, these results highlight the critical role of the sesquiterpene lactone core, and the observed enhanced combined activity between costunolide and reduced glutathione is consistent with a possible contribution of the NRF2/glutathione antioxidant axis to its anti-DENV action. Overall, this study highlights *L. nobilis* as a previously underexplored source of antiviral sesquiterpene lactones and establishes costunolide (**2**) as a promising lead against HCV and DENV, warranting further investigation across additional Flaviviridae members.

## 1. Introduction

*Laurus nobilis* L. (family Lauraceae), commonly known as bay, sweet bay, true laurel, or Roman laurel, is an evergreen aromatic tree widely distributed throughout the Mediterranean region and Europe. Its dried leaves are commonly used as a spice in culinary applications and are also incorporated into perfumes and soaps in the cosmetics industry. In traditional medicine, *L. nobilis* has been employed for its stomachic and carminative properties, as well as for the treatment of rheumatism, earaches, gastrointestinal inflammation, and various gastric disorders [1,2]. Previous studies have demonstrated a wide range of biological activities associated with *L. nobilis* leaves, including antimicrobial, antioxidant, wound-healing, cytotoxic, and trypanocidal effects [3,4,5,6,7]. Laurel leaves contain a wide variety of phytochemicals that can be broadly classified into volatile and non-volatile compounds. The volatile fraction is dominated by essential oils rich in monoterpenes and sesquiterpenes (e.g., 1,8-cineole, sabinene, and linalool), which are responsible for the plant’s characteristic aroma. The non-volatile constituents include several important classes: sesquiterpene lactones and other sesquiterpenoids, which are characteristic compounds of laurel leaves; alkaloids of aporphine and noraporphine types; and norisoprenoids (megastigmane derivatives). Additionally, laurel leaves are rich in phenolic compounds, including simple phenolics (such as caffeic and ferulic acids), flavonoids (e.g., quercetin, kaempferol, and apigenin glycosides), and proanthocyanidins, which are important for antioxidant activity. Other minor constituents include tocopherols (vitamin E compounds), as well as sugars, organic acids, and polysaccharides [8].

Hepatitis C virus (HCV) infection remains a significant global public health challenge and is a leading cause of chronic liver disease, often progressing to liver cirrhosis and hepatocellular carcinoma. A notable feature of HCV infection is its typically asymptomatic nature, which allows the virus to remain undetected for decades before culminating in severe complications such as chronic liver failure or malignancy. In recent years, the advent of direct-acting antivirals (DAAs), particularly in combination regimens, has substantially improved treatment outcomes and cure rates among HCV-infected individuals. Despite these advances, the high cost of DAA therapy and limited accessibility in many regions continue to restrict their global impact. Furthermore, the emergence of drug-resistant viral variants poses an ongoing challenge to treatment efficacy (World Health Organization (WHO); Hepatitis C, 2026). Similarly, dengue fever represents another major viral disease of global concern and is classified by the World Health Organization as a neglected tropical disease. It is caused by the dengue virus (DENV), a member of the *Flaviviridae* family and *Orthoflavivirus* genus. DENV is an enveloped, single-stranded, positive-sense RNA virus transmitted to humans through the bite of infected *Aedes* mosquitoes. Following transmission, the virus primarily targets immune cells, including dendritic cells, macrophages, monocytes, and lymphocytes. Dengue poses a substantial global health burden, with nearly half of the world’s population living in areas at risk of transmission and an estimated 100–400 million infections occurring annually. Although most infections are mild, severe dengue can result in life-threatening complications with mortality rates reaching up to 20% in untreated cases. The absence of specific and widely effective antiviral therapies underscores the urgent need for the development of novel therapeutic strategies [9,10,11].

Sesquiterpene lactones (STLs) constitute one of the largest and most structurally diverse classes of natural products, widely distributed in medicinal plants and recognized for their broad spectrum of biological activities. In recent years, accumulating evidence has highlighted the potential of STLs as promising candidates for the treatment of various viral infections. Their diverse chemical scaffolds and unique mechanisms of action make them attractive lead compounds for antiviral drug discovery. Despite these encouraging findings, the antiviral potential of STLs remains relatively underexplored, warranting further investigation into their therapeutic applications. Building on this premise, the present study focuses on the isolation and evaluation of bioactive STLs from *Laurus nobilis*. Two major sesquiterpene lactones were successfully isolated and assessed for their antiviral activity against hepatitis C virus (HCV) and dengue virus (DENV). Notably, both compounds exhibited significant antiviral effects, underscoring their potential as promising lead candidates for the development of novel antiviral agents targeting these clinically important viruses.

## 2. Material and Methods

### 2.1. General Experimental Procedures

Organic solvents were purchased from Wako Pure Chemical Industries (Osaka, Japan). Analytical TLC was performed on precoated silica gel 60 GF_254_ (20 × 20 cm × 0.2 mm thick) aluminium-backed plates from Merck Co. (Darmstadt, Germany). Nuclear Magnetic Resonance spectra were recorded on a Bruker Avance III HD (400 MHz) spectrometer (Billerica, MA, USA). Deuterated chloroform (CDCl_3_) was used as the NMR solvent. Chemical shifts were measured in parts per million (ppm) using tetramethylsilane (TMS) as an internal standard. High-resolution mass spectrometry (HRMS) was performed with an Agilent 6545 Q-TOF mass spectrometer (Agilent Technologies, Santa Clara, CA, USA).

### 2.2. Plant Material

Dried leaves of *Laurus nobilis* (100 g), in whole form, were purchased as a commercial product originating from China and distributed by Kobe Bussan Co., Ltd. (Hyogo, Japan).

### 2.3. Extraction and Isolation Procedures

Dried powdered leaves of *Laurus nobilis* (100 g) were exhaustively defatted with *n*-hexane using a Soxhlet apparatus (continuous extraction for 6–8 h). The *n*-hexane extract was concentrated under reduced pressure to afford the *n*-hexane-soluble fraction (5 g), while the defatted plant material was retained for further processing. The defatted residue was air-dried, then alkalinized with 28% ammonium hydroxide (NH_4_OH) using a sufficient volume to uniformly moisten the powder. The mixture was thoroughly homogenized and allowed to dry at room temperature. The alkalinized material was subsequently subjected to successive Soxhlet extraction with dichloromethane (DCM) and methanol (MeOH) (approximately 6 h each). The solvents were removed under reduced pressure to yield DCM-soluble (1.7 g) and MeOH-soluble (8.5 g) fractions. The isolation of the target compounds was carried out from the *n*-hexane-defatted portion.

The *n*-hexane-defatted extract (5 g) was subjected to medium-pressure liquid chromatography (MPLC) using a Biotage^®^ Selekt flash chromatography system (Biotage, Uppsala, Sweden) equipped with a Biotage^®^ Sfär HC Duo silica column (50 g). Separation was performed under normal-phase conditions using *n*-hexane (solvent A) and ethyl acetate (EtOAc, solvent B) as the mobile phase. Elution was achieved using a stepwise gradient starting with 100% *n*-hexane (0% EtOAc), followed by a gradual increase in polarity as follows: 0–10% EtOAc, 10–20%, 20–30%, 30–40%, 40–50%, 50–60%, 60–70%, 70–80%, 80–90%, and finally 90–100% EtOAc, with each step maintained for 5 column volumes (CV). A final isocratic hold at 100% EtOAc was applied for 10 CV. Chromatography was performed at a flow rate of 120 mL/min with UV detection monitored over 200–400 nm (with channels at 210 and 254 nm). A total of 20 fractions (1–20H) were collected during the chromatographic separation, based on their chromatographic profiles and UV absorption signals.

Fraction 6H (1236.2 mg), eluted with 10–20% EtOAc/*n*-hexane, was further subjected to MPLC (Pure C-850 FlashPrep^®^, Büchi, Flawil, Switzerland) on a normal-phase silica column. Separation was carried out on a prepacked silica flash column (24 g) using a gradient of *n*-hexane–EtOAc (100:0 → 80:20) at a flow rate of 32 mL/min. Elution was monitored by ELSD and DAD at 210 and 254 nm, affording several subfractions. Subfractions were collected in sequential tubes (typically 25 mL per tube) and combined based on thin-layer chromatography (TLC) profiling (silica gel plates, UV 254/365 nm detection and 7% H_2_SO_4_/MeOH spray reagent). This separation yielded 59 subfractions, of which subfractions 3–10 (437 mg) was selected for further purification. This subfraction (3–10) was purified by repeated MPLC on a normal-phase silica column using an isocratic elution of *n*-hexane–EtOAc (95:5) on a prepacked normal phase flash column (12 g), and a flow rate of 20 mL/min. using ELSD and DAD (210, 254 nm) detectors to yield 44 subfractions (1–44). Among these, subfractions 29 and 31 were selected for the isolation of the target compounds.

Subfraction 29 (24.7 mg) was further purified by preparative MPLC on a normal-phase silica preparative column (Büchi HPLC column, silica, 10 µm, 250 × 21.2 mm) using an isocratic system of *n*-hexane–EtOAc (95:5) at a flow rate of 8 mL/min, with detection by ELSD. Fractions were collected and monitored by TLC, leading to the isolation of a pure compound (9.9 mg), designated as **1**.

Subfraction 31 (44.2 mg) was obtained as a single spot on TLC and was directly assigned as compound **2** without further purification. The isolated compounds **1** and **2** were dried under reduced pressure and subjected to spectroscopic analysis for structural elucidation.

### 2.4. Bioassays

#### 2.4.1. Cell Culture

The Huh5-2 cell line stably carrying the subgenomic HCV reporter replicon I389luc-ubi-neo/NS3-3′/Con1/5.1 (genotype 1b, Con1 strain) was used for the evaluation of the anti-HCV activity [12]. For the assessment of anti-DENV activity, we used the Huh7-D2 stable cell line (kindly provided by C. M. Rice, The Rockefeller University, New York, NY, USA), which contains a DENV-2 (strain 16681) subgenomic replicon generated by replacing the structural protein-coding region downstream of the 5′ cyclization sequence with a humanized Renilla luciferase (R-Luc) reporter cassette, allowing quantification of viral RNA replication through R-Luc activity [13]. Huh7-Lunet, Huh5-2 cells (kindly provided by Ralf Bartenschlager), Huh7-D2 and Huh7.5 (contributed by Prof. C. Rice) were cultured at 37 °C and 5% CO_2_, in high-glucose (25 mM) DMEM (Invitrogen, Waltham, MA, USA) supplemented with 10% (*v*/*v*) fetal calf serum, 2 mM L-glutamine, 100 U/mL penicillin, 100 µg/mL streptomycin, and 0.1 mM non-essential amino acids. To maintain a stable HCV replicon expression, the culture medium was supplemented with 500 µg/mL geneticin (G418).

#### 2.4.2. Viruses

For the production of DENV, the plasmid pFK-DVR2A (based on the DENV-2 strain 16681) [14], was linearized with XbaI and used as a template for in vitro RNA transcription as previously reported. RNA yield and purity was assessed by Nanodrop spectrophotometry, and integrity was confirmed by denaturing agarose gel electrophoresis. Full-length viral RNA was electroporated into VeroE6 cells [15]. At 24 h post-electroporation, the medium was replaced with complete DMEM supplemented with 15 mM HEPES (pH 7.5), and cells were incubated until the appearance of virus-induced cytopathic effects. Culture supernatants were harvested between days 4 and 7 post-transfection, pooled, clarified through a 0.45 μm filter, aliquoted, and stored at −80 °C. Viral stocks were amplified by passaging onto fresh VeroE6 cells, and titers were determined by standard plaque assay following 4 h infection [16].

The plasmid pHCV N.2/GLuc2A, encoding the full-length hepatitis C virus (HCV) genotype 1b (HCV-N) reporter genome, was used as a template for in vitro RNA transcription [17]. RNA yield and purity were assessed by NanoDrop spectrophotometry, and RNA integrity was verified by denaturing agarose gel electrophoresis. Full-length viral RNA was electroporated into cultured cells following previously established protocols [15]. Briefly, 4 × 10^6^ cells were detached by trypsinization, resuspended in Cytomix buffer [18] supplemented with 2 mM ATP and 5 mM glutathione, mixed with 10 μg of viral RNA, and subjected to electroporation using a Gene Pulser electroporation system (Bio-Rad, Hercules, CA, USA). Immediately after electroporation, cells were transferred into 20 mL of complete DMEM and seeded into culture plates according to the requirements of each experimental setup.

#### 2.4.3. Alamar Blue Assay for Cell Viability

Compound cytotoxicity was evaluated using the AlamarBlue^®^ viability assay (Sigma-Aldrich, St. Louis, MO, USA). Huh7 cells (10^4^ cells/well) were seeded in 96-well plates and, after 24 h, treated with 3-fold serial dilutions of the compounds (highest concentration tested: 200 μM) or DMSO (control) for 72 h at 37 °C and 5% CO_2_. AlamarBlue^®^ reagent (10 μL) was added after 10 μL of medium was removed, and plates were incubated for 4 h before absorbance was measured at 570 and 600 nm at 0 h and 4 h. Cell viability was calculated and compared to the control cells, and the CC_50_ values were calculated by nonlinear regression analysis of log-transformed concentrations using GraphPad Prism 9.0 (GraphPad Software Inc., San Diego, CA, USA).

#### 2.4.4. Cell-Based Antiviral Activity Assessment by Luciferase Reporter Assay

The antiviral potency of the tested compounds was evaluated in Huh5-2 HCV replicon cells, in Huh7-Lunet cells electroporated with pHCV N.2 RNA, in Huh7-D2 cells stably carrying the DENV-2 subgenomic replicon (serotype 2, strain 16681), and in DENV-infected Huh7.5 cells. For DENV infection assays, Huh7.5 cells were inoculated with DVR2A for 4 h, after which the inoculum was replaced with complete DMEM. Compounds and the vehicle control (DMSO) were applied in serial dilutions and incubated for 72 h (HCV) or 48 h (DENV). Antiviral activity was quantified by measuring virus-encoded luciferase reporter activity.

Firefly luciferase (F-Luc) and Renilla luciferase (R-Luc) activity was determined in cell lysates using the Luciferase Assay System (Promega Corporation, Madison, WI, USA), following the manufacturer’s protocol. Gaussia luciferase (G-Luc) activity was quantified in collected cell supernatants by adding coelenterazine (Promega Corporation, Madison, WI, USA) at a final concentration of 12 µM, prepared in assay buffer containing 500 mM NaCl, 50 mM potassium phosphate, and 1 mM EDTA (pH 7.4). Luminescence measurements were performed using an GloMax 20/20 single-tube luminometer (Promega Corporation, Madison, WI, USA), with each sample measured for 10 s. Luciferase activity values were normalized to total protein content determined using the Bradford protein assay (Bio-Rad, Hercules, CA, USA).

Half-maximal effective concentrations (EC_50_), defined as the compound concentration that produces a 50% reduction in luciferase signal, were determined by nonlinear regression analysis using GraphPad Prism 9.0. Selectivity indices (SI) were calculated as the ratio of CC_50_ to EC_50_. Compounds with EC_50_ > 10 µM were considered non-effective; therefore, the higher concentration used in the activity assays was 10 µM.

#### 2.4.5. Western Blot Analysis

Western blotting was performed as described elsewhere [19]. The following antibody dilutions were used: 1:4000 for the HCV NS5A (9E10) monoclonal antibody, 1:3000 for the DENV NS3 monoclonal antibody (GeneTex International Corporation, Hsinchu City, Taiwan) and 1:6000 for the β-actin monoclonal antibody (Merck-Millipore, Rahway, NJ, USA).

#### 2.4.6. Total RNA Extraction and Quantification of Viral Replicons

Total RNA was extracted from cells using TRIzol reagent (Thermo Fisher Scientific, Waltham, MA, USA) according to the manufacturer’s instructions. Replicon RNA abundance was subsequently assessed by reverse transcription (RT) followed by quantitative real-time PCR (qPCR). cDNA synthesis was performed using Moloney Murine Leukemia Virus (MMLV) reverse transcriptase (Promega, Madison, United States) with gene-specific reverse primers designed to detect the Con1 IRES region (Con1-IRES-R: 5′-GGATTCGTGCTCATGGTGCA-3′), the DENV IRES region (DV-A: 5′-ACCATTCCATTTTCTGGCGTT-3′), and the endogenous control gene YWHAZ (YWHAZ-R: 5′-GGATGTGTTGGTTGCATTTCCT-3′). Quantitative PCR was carried out using KAPA SYBR FAST qPCR Master Mix (Sigma-Aldrich, St. Louis, MO, USA) with the following primer pairs: Con1 IRES-specific primers (Con1-IRES-F: 5′-GGCCTTGTGGTACTGCCTGATA-3′ and Con1-IRES-R), DENV IRES-specific primers (DV-S: 5′-GAAAGACCAGAGATCCTGCTGTCT-3′ and DV-A), and YWHAZ primers (YWHAZ-F: 5′-GCTGGTGATGACAAGAAAGG-3′ and YWHAZ-R). The transcript levels of YWHAZ were used as an internal reference for normalization.

#### 2.4.7. Statistical Analysis

The results obtained are presented as bar graphs. Bars correspond to mean values from at least three independent experiments performed in triplicate. Error bars represent the standard deviation (SD). The statistical analysis of the results was carried out using One-Way ANOVA, followed by the appropriate post hoc analysis.

## 3. Results

### 3.1. Chemistry

The phytochemical investigation of *Laurus nobilis* L. leaves led to the isolation of two major sesquiterpene lactones, designated as compounds **1** and **2** (Figure 1). Their structures were elucidated using HRMS and 1D NMR (^1^H and ^13^C) spectroscopic analyses, and further confirmed by comparison with previously reported data. Compound **1** was identified as dehydrocostus lactone, while compound **2** was characterized as costunolide. Both compounds belong to the sesquiterpene lactone class and are representative constituents of *L. nobilis*. The high purity (>98%) of the isolated compounds was confirmed by spectroscopic analysis, supporting their suitability for further studies.

**Compound 1** was isolated as a white to pale yellow powder, $\left[\alpha \right]\begin{array}{c} 25\\ D \end{array}$ −9.0 (c 0.2, CHCl_3_). Its molecular formula was established as C_15_H_18_O_2_ by positive-mode HRMS, which exhibited a quasi-molecular ion peak at *m*/*z* 231.1377 [M + H]^+^ (calcd. for C_15_H_19_O_2_^+^, 231.1385). The data are consistent with a sesquiterpene framework and were further supported by the corresponding ^1^H and ^13^C NMR spectra (Supplementary Figures S1–S3). The ^13^C NMR spectrum (Table 1) displayed 15 distinct carbon resonances, confirming the sesquiterpenoid nature of the compound. The downfield signal at δ_C_ 170.3, corresponding to a carbonyl carbon, along with the olefinic signals at δ_C_ 120.2 and 151.3 were characteristic for an α,β-unsaturated γ-lactone moiety [20]. Additionally, multiple signals distributed in the olefinic region (δ_C_~109–151 ppm) indicated a high degree of unsaturation within the molecule. The ^1^H NMR spectrum provided further structural insights, showing diagnostic olefinic proton signals at δ_H_ 5.51 and 6.23 (each 1H, d, *J* ≈ 3 Hz), which are indicative of an exocyclic double bond conjugated to a carbonyl group. Additional resonances at δ_H_ 4.83–4.92 and 5.09–5.29 were consistent with exocyclic methylene protons, further supporting the presence of multiple terminal double bonds. The remaining proton signals, appearing predominantly in the aliphatic region (δ_H_ 1.2–3.0), reflect a complex and highly substituted carbon framework typical of sesquiterpene lactones. The combination of a lactone carbonyl signal, multiple olefinic resonances, and characteristic exocyclic methylene proton signals strongly suggests a guaianolide-type sesquiterpene skeleton. From the spectral analysis and comparisons with the literature [20,21,22], the structure of compound **1** was characterized as dehydrocostus lactone, a sesquiterpene lactone of the guaianolides class.

**Compound 2** was obtained as colorless needles, $\left[\alpha \right]\begin{array}{c} 25\\ D \end{array}$ 127.0 (c 0.3, CHCl_3_). Its molecular formula was determined to be C_15_H_20_O_2_ on the basis of positive-mode HRMS, which showed a molecular ion peak at *m*/*z* 233.1535 [M + H]^+^ (calcd. for C_15_H_21_O_2_^+^, 233.1542). The proposed molecular composition was in full agreement with the recorded ^1^H and ^13^C NMR spectral data (Supplementary Figures S4–S6). The ^13^C NMR spectrum (Table 1) revealed 15 carbon signals, including a characteristic resonance at δ_C_ 170.5, assignable to a lactone carbonyl carbon. Additional carbon signals in the olefinic region (δ_C_ ~119–141 ppm) suggested the presence of conjugated double bonds within the molecule. The ^1^H NMR spectrum displayed two olefinic proton signals at δ_H_ 5.54 and 6.27 (each ^1^H, d, *J* = 3.24 Hz), characteristic of an exocyclic methylene group conjugated with the lactone carbonyl. A notable structural feature distinguishing compound **2** from compound **1** is the presence of two methyl group signals at δ_H_ 1.44 (3H, s) and 1.72 (3H, d, *J* = 1.44 Hz), consistent with methyl substituents on an unsaturated carbon framework. In addition, several proton resonances observed over the range δ_H_ 1.6–4.8 reflected a substituted and conformationally flexible sesquiterpene backbone. The overall spectroscopic pattern, including the lactone carbonyl, olefinic signals, and methyl group resonances, was characteristic of a germacranolide-type sesquiterpene lactone. A comprehensive comparison of the obtained NMR data with previously reported values [20,21,22,23,24] confirmed the identity of compound **2** as costunolide.

### 3.2. Biological Evaluation

The antiviral efficacy of compounds **1** and **2** against HCV were evaluated in Huh5-2 cells, which stably express the HCV genotype 1b (Con1) subgenomic replicon together with the firefly luciferase reporter gene (F-luc), the activity of which serves as a quantitative surrogate for viral replication. Prior to their induction into the antiviral assays the cytotoxicity of both compounds was determined in Huh7 cells using Alamar Blue reagent. Antiviral activity was quantified as relative luminescence units (RLU) normalized to total protein content. For both compounds, Half-Maximal Effective Concentrations (EC_50_) and half-maximal cytotoxic concentrations (CC_50_) were determined using non-linear regression analysis, and selectivity indices (SI = CC_50_/EC_50_) were calculated to define the safety margin. As presented in Table 2 and Figure 2, both derivatives inhibited HCV replication in a dose-dependent manner, yielding EC_50_ values of 2.34 μM and 2.06 μM, and SI values of 14.31 and 13.88, respectively.

Moreover, to elucidate the underlying mechanism of action, we investigated whether the potency of these compounds is restricted to the replication stage of the HCV life cycle or is more pronounced when assessed using full-length viral constructs. Specifically, an in vitro–transcribed full-length HCV genotype 1b genome (HCV-N2), which concomitantly expresses a Gaussia luciferase reporter, was introduced into Huh7-Lunet cells via electroporation. Notably, compound 2 exhibited stronger suppression of HCV propagation, yielding an EC_50_ value of 1.11 μM (SI = 26) whereas compound **1** showed no significant change in efficacy under these conditions (Figure 3A,B). The antiviral capacity of compound **2** was further validated by Western blot analysis of NS5A protein levels (Figure 3C). To further explore its translational potential, we assessed the combinatory effect of compound **2** with daclatasvir, a clinically approved NS5A inhibitor [25], and observed an augmented suppression of HCV propagation (Figure 3D), supporting the potential of compound **2** as an adjunct to existing direct-acting antiviral regimens.

Given the promising anti-HCV activity of compound **2**, its antiviral potential was next evaluated against dengue virus (DENV), a major global health threat for which no clinically approved therapeutics exist. Given that compound **2** exhibited stronger inhibition of full-length HCV compared to the subgenomic replicon, DENV assays were performed using both replicon and infectious models. Ribavirin was used in parallel in order to ensure efficiency of the antiviral assays, yielding an EC_50_ value of 15 μM. First, to define the stage of the DENV life cycle targeted by compound **2**, four mechanistically informative treatment conditions were employed in our infectious system. Viral entry was evaluated either by pre-treating Huh7.5 cells with serial dilutions of the compound for 2 h prior to infection (Figure 4A), or by supplementing the viral inoculum with the same concentrations during infection (Figure 4B). In addition, the effect of the compound on virion stability was assessed by incubating the virus supernatant with the compound for 2 h before infection (Figure 4C). Finally, post-entry activity was examined by administering the compound **2** h after infection and maintaining the cultures for an additional 48 h (Figure 4D). Antiviral efficacy was quantified by measuring relative luciferase activity normalized to total protein content. As shown in Figure 4, the most pronounced inhibitory effect of compound **2** occurred during the post-infection phase (EC_50_ = 1.27 μM, SI = 22.5), indicating interference with DENV replication and/or viral egress. Pre-treatment of cells did not affect viral propagation (EC_50_ > 20 μM). Co-treatment with virus and compound yielded an EC_50_ of 3.71 μM, consistent with early replication inhibition. A modest reduction in virion stability was observed, reflected by a two-fold increase in EC_50_ relative to the co-treatment condition (EC_50_ = 7.91 μM). Next, the effect of compound 2 on viral replication was validated in Huh7-D2 replicon cells, yielding an EC_50_ value of 5.89 μM (Table 2).

The antiviral profile of costunolide (compound **2**) against DENV was further examined by quantifying its effects on viral RNA accumulation and protein expression (Figure 5A,B). Given that the dominant suppression of DENV propagation was observed when the compound was administered post-infection, this treatment condition was selected for subsequent analyses. Consistent with the luciferase-based assays, compound **2** reduced both viral RNA and (Figure 5A) protein levels (Figure 5B) with EC_50_ values of approximately 1.4 μM across the two experimental readouts, confirming that its principal antiviral activity occurs during post-entry stages of the DENV life cycle.

DENV infection is known to disrupt cellular redox homeostasis and suppress NRF2-driven antioxidant defenses [26,27]. Costunolide, on the other hand, has been reported to counteract cellular oxidative stress by inducing NRF2 signaling and enhancing downstream antioxidant responses in other biological systems [28,29]. This raises the possibility that a related redox-modulating mechanism could contribute to its antiviral activity. To explore this possibility at a preliminary, pharmacological level, we introduced reduced glutathione (GSH), a well-characterized antioxidant which could justify the observed antiviral activity. To further investigate this, we also introduced reduced glutathione (GSH), a well-characterized antioxidant [30], to examine any possible combinatory effect between the two compounds. As anticipated, GSH alone reduced DENV propagation (Figure 5B). Moreover, combined treatment with compound 2 and GSH produced a more pronounced antiviral effect. This was confirmed by determining the coefficient of drug interaction, which was calculated at 0.74 (CDI = AB/(A × B), where A and B represent the effects of the individual treatments with each compound, while AB corresponds to their combination).

## 4. Discussion

The biological evaluation of the isolated STLs revealed promising low-micromolar antiviral activity (${\mathrm{EC}}_{50}<10\;\mu M$) against HCV and DENV replication. Within the broader landscape of natural antiviral discovery, several terrestrial plant-derived secondary metabolites have been identified as highly potent leads displaying sub-micromolar to low-micromolar efficacy against positive-sense single-stranded RNA viruses. For instance, lycorine, an Amaryllidaceae alkaloid, exhibits strong anti-flaviviral activity against DENV-2 and Zika virus replication [31]. Similarly, silibinin (a flavonolignan from *Silybum marianum*) inhibits HCV entry and viral RNA synthesis [32], while epigallocatechin gallate (EGCG) from *Camellia sinensis* blocks early cell entry across major HCV genotypes [33]. Furthermore, curcuminoids display marked anti-DENV replication capabilities within sub-micromolar to low-micromolar ranges [34].

The low-micromolar potency demonstrated by the *Laurus nobilis* sesquiterpene lactones aligns well with these established terrestrial plant leads, reinforcing the potential of α,β-unsaturated γ-lactone frameworks as viable scaffolds for novel antiviral drug development.

Sesquiterpene lactones (STLs) have emerged as a valuable class of natural compounds with broad-spectrum antiviral activity, attributed to their remarkable structural diversity and the presence of key electrophilic pharmacophores such as the α-methylene-γ-lactone moiety. These compounds exert antiviral effects through multiple mechanisms, including inhibition of viral entry, suppression of viral RNA replication, interference with viral protein synthesis, and modulation of host–virus interactions, thereby targeting several critical stages of the viral life cycle [35,36,37,38]. The antiviral activity of STLs spans a wide range of viruses, including hepatitis C virus (HCV), hepatitis B virus (HBV), herpes simplex virus (HSV), influenza viruses, and emerging pathogens such as SARS-CoV-2. Structurally, STLs are categorized into several subclasses, such as guaianolides, germacranolides, eudesmanolides, and xanthanolides, each contributing differently to antiviral efficacy. For example, guaianolides like grosheimol and cynaropicrin exhibit strong inhibitory activity against multiple HCV genotypes through interference with viral entry, while germacranolides such as parthenolide demonstrate potent antiviral effects against HCV, HSV-1, and SARS-CoV-2. Additionally, xanthanolides such as xanthatin display broad-spectrum activity against several RNA and DNA viruses, including herpes simplex, vaccinia, and vesicular stomatitis viruses, further emphasizing the versatility of STLs as antiviral scaffolds. The biological activity of these compounds is strongly influenced by their chemical features, particularly the presence of reactive α,β-unsaturated carbonyl systems that enable covalent interaction with viral or host proteins via Michael addition, thereby disrupting essential viral processes [35,37].

Among the extensively studied STLs, dehydrocostus lactone and costunolide have demonstrated significant and broad antiviral activities. Dehydrocostus lactone is a guaianolide-type STL that exhibits potent inhibitory effects against multiple viruses, including HBV, HCV, HSV-1, and hepatitis A virus (HAV). It significantly suppresses hepatitis B surface antigen (HBsAg) production in Hep3B cells with an IC_50_ value of approximately 2 μM, highlighting its strong anti-HBV activity. In addition, it shows notable anti-HCV activity with an EC_50_ value of 3.08 ± 0.60 μM in subgenomic replicon systems, suggesting its ability to interfere with viral replication. Dehydrocostus lactone also exhibits considerable antiviral activity against HSV-1, achieving inhibition rates exceeding 85%, and demonstrates moderate effects against HAV. Mechanistically, molecular docking studies propose that its antiviral action may involve binding to key viral enzymes, such as HSV-1 DNA polymerase and HAV 3C protease, thereby blocking viral replication and protein processing. Its broad antiviral spectrum is likely related to the presence of an exo-methylene lactone group, which enhances its reactivity and interaction with biological targets [35,39,40,41,42].

Similarly, costunolide, a germacranolide-type STL, displays potent antiviral activities against several clinically relevant viruses. It exhibits strong anti-HBV activity, inhibiting HBsAg production in Hep3B cells with an IC_50_ value of approximately 1 μM and significantly reducing viral mRNA expression levels. Furthermore, costunolide demonstrates notable anti-HCV activity, with EC_50_ values in the low micromolar range (approximately 2.69 ± 0.57 μM), indicating effective suppression of viral RNA replication. In addition to hepatitis viruses, costunolide also exhibits antiviral effects against HSV-1 and HAV, with measurable inhibition percentages, although its activity against HAV appears less pronounced. Its antiviral mechanism has been linked to its ability to interfere with viral gene expression and replication processes, as well as its interaction with key cellular signaling pathways. Importantly, costunolide, along with related compounds such as parthenolide, has been shown to exert dose-dependent inhibition of HCV RNA replication, and its activity can be enhanced when used in combination with interferon-α, suggesting potential for combination therapy [35,39,40,41,42,43].

STLs have been only marginally explored for their antiviral activity against DENV, with available evidence indicating that their potential remains underinvestigated. An early study by Borgo et al. [44] demonstrated that, among several STLs isolated from Asteraceae species, only 2-oxo-8-deoxyligustrin and santhemoidin C exhibited moderate anti-DENV-2 activity (EC_50_ ≈ 3 µM), suggesting that antiviral effects are highly structure-dependent rather than a general feature of this class. More recent findings further highlight this structure–activity relationship, as the modified STL desacyl grazielia acid tiglate displayed significantly enhanced potency (EC_50_ = 2.36 µM) with very high selectivity, surpassing previously reported STLs. Collectively, these studies indicate that while intrinsic anti-DENV activity in STLs may be limited, targeted structural modifications can markedly improve efficacy, supporting their potential as promising lead compounds for antiviral drug development [45].

Our findings indicate that both dehydrocostus lactone **1** and costunolide **2** exhibit notable antiviral activity against HCV, as assessed using subgenomic replicon cells and infectious viral constructs. Both compounds suppressed viral propagation in the low micromolar range. Costunolide **2** exhibited greater potency in the full-length HCV system than in the subgenomic replicon, suggesting that its antiviral activity extends beyond RNA replication to additional stages of the viral life cycle. Moreover, compound **2** showed cooperative activity with daclatasvir (DCV), a well-characterized NS5A inhibitor, providing initial insight into its underlying mechanism of action. These findings, together with the favorable balance between antiviral activity and cytotoxicity, highlight costunolide **2** as a potent candidate for incorporation into therapeutic regimens as part of HCV elimination initiatives.

In contrast, among the two compounds studied, only costunolide **2** retained an appreciable antiviral activity against another member of the Flaviviridae family, DENV. Specifically, in DENV replicon cells, compound **2** yielded an EC_50_ value of 5.89 ± 0.22 µM and a CC_50_ greater than 20 µM, resulting in a moderate selectivity index of 3.4. Notably, as in the case of HCV, the activity of **2** was markedly increased (EC_50_ = 1 µM; a 5-fold reduction compared with the replicon system) in DENV-infected Huh7.5 cells.

The increase in the potency (EC_50_ and SI) of Costunolide **2** in the infectious-virus models compared to the replicon systems, likely reflect the inherent limitations of subgenomic replicons, which reconstitute only the RNA-replication stage of the viral life cycle and lack the structural proteins required for virion assembly, egress, and subsequent rounds of infection [14,46]. Infectious cell models capture additional stages of the viral life cycle and host-response aspects that the compound **2** may affect, including host oxidative-stress responses, and are absent from the replicon system. Thus, they provide a more physiologically relevant context in which the full antiviral potential can be detected. This could explain the modest SI values observed in the replicons, as compared to the infectious assays. Moreover, for early natural-product hits, even SI values in the low single digits (SI < 10) are routinely reported and considered informative starting points for structure–activity exploration [47].

Concerning DENV, Costunolide exerted its most pronounced activity under post-infection treatment conditions. This analogue has previously been reported to activate NRF2 signaling and enhance antioxidant responses in other experimental systems [28,48], raising the possibility that a similar mechanism may contribute to its anti-DENV effects. The combined antiviral activity observed between costunolide (**2**) and reduced glutathione (CDI = 0.74; see above) is consistent with, but does not by itself demonstrate, NRF2 pathway activation or dependence. Definitive attribution of the compound’s antiviral properties to NRF2-mediated mechanisms will require dedicated follow-up studies, including assays of NRF2 nuclear translocation, antioxidant-response-element reporter activity, and quantification of NRF2 target gene and protein expression following compound treatment.

The results presented on the anti-HCV activity of dehydrocostus lactone **1** and costunolide **2** are in agreement with previously reported work [41,42]. The principal novelty of the present study therefore lies in the newly characterized anti-DENV activity of costunolide and in the mechanistic evidence linking this activity to NRF2/glutathione-mediated antioxidant signalling, rather than in the discovery of an entirely new antiviral chemotype.

## 5. Conclusions

The present study reveals *L. nobilis* as a rich and promising source of antiviral sesquiterpene lactones with significant therapeutic potential. Both dehydrocostus lactone **1** and costunolide **2** demonstrated potent inhibition of HCV replication at low micromolar concentrations, coupled with favorable selectivity, highlighting their strong potential as anti-HCV agents. Notably, costunolide **2** also exhibited measurable activity against DENV serotype **2**, indicating that its antiviral effect is not restricted to HCV, while dehydrocostus lactone 1 appeared more selectively active against HCV (genotype 1b). These findings emphasize the critical role of structural features in modulating antiviral activity within this compound class. The involvement of the NRF2/glutathione antioxidant axis in costunolide’s anti-DENV activity is currently supported by indirect pharmacological evidence (enhanced combined activity with exogenous GSH) as a working hypothesis. Collectively, the results position sesquiterpene lactones, particularly costunolide, as attractive and versatile lead scaffolds for antiviral drug discovery, meriting deeper mechanistic studies, including direct assessment of NRF2 pathway activation, and targeted structural optimization to unlock their full therapeutic potential.

## Figures and Tables

**Figure 1:**
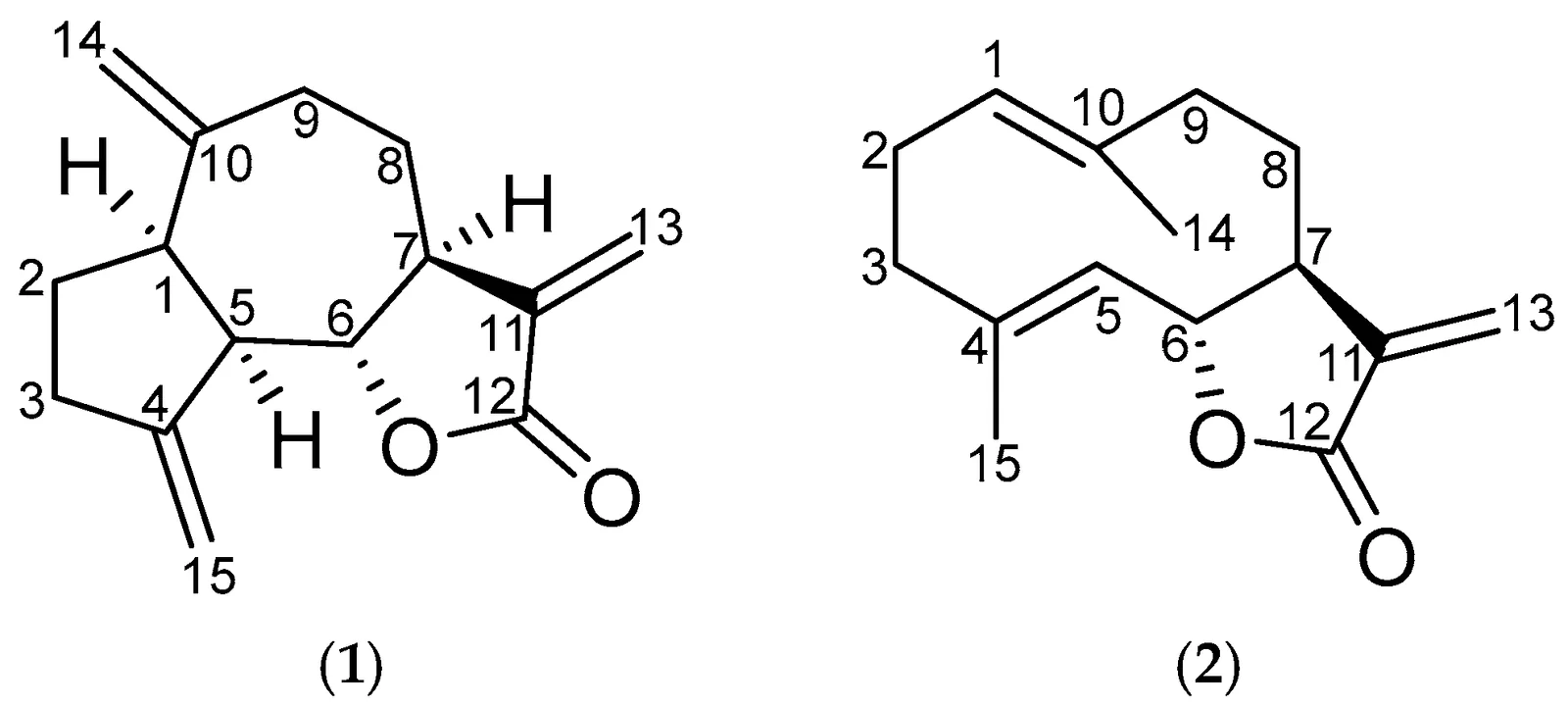
The isolated compounds **1** and **2** from *Laurus nobilis* leaves.

**Figure 2:**
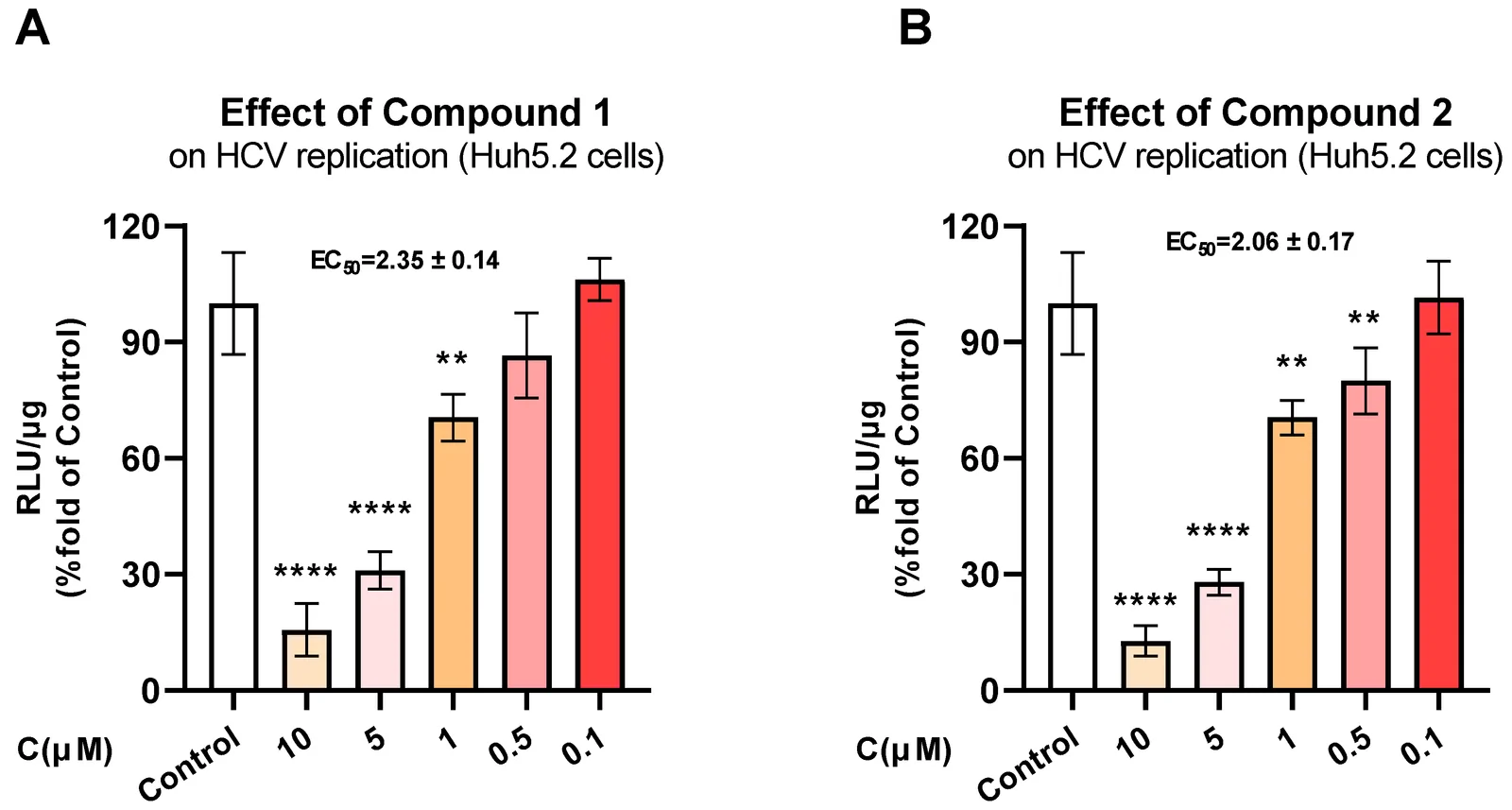
The inhibitory effect of compounds **1** (**A**) and **2** (**B**) on HCV replication (Huh5-2 cells, HCV-genotype 1b replicon cells). Huh5-2 were treated for 72 h with serial dilutions of the compounds or were mock-treated DMSO (Control). Firefly luciferase activity was measured and expressed as RLU/μg of total protein content. Data are presented as means ± SD from three independent experiments in triplicate. Statistical analysis of the results was carried out using one-way ANOVA, followed by Bonferroni correction test. ** *p* < 0.01, **** *p* < 0.0001 vs. control.

**Figure 3:**
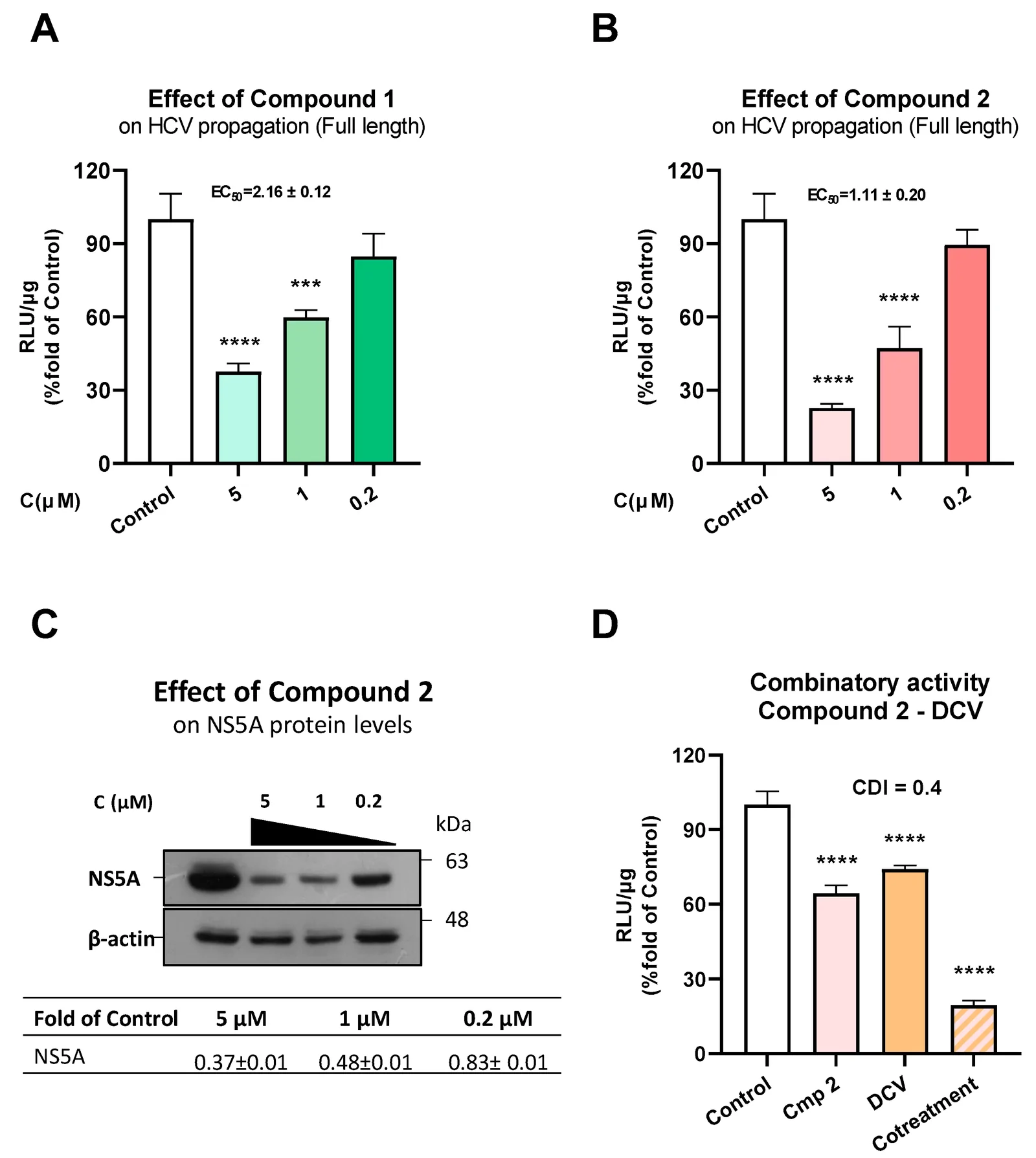
The inhibitory effect of compounds **1** (**A**) and **2** (**B**) at the level of HCV replication in Huh7-Lunet cells electroporated with the pHCV-N/GLuc2A reporter genome. (**A**,**B**) Measurement of Gaussia luciferase (G-Luc) activity in cell culture supernatants. Luciferase activity was measured 72 h post-electroporation and expressed as percentage of DMSO-treated control cells. Statistical analysis was performed using One-Way ANOVA followed by the appropriate post hoc analysis. *** *p* < 0.001, **** *p* < 0.0001 vs. control. (**C**) Western blot analysis of HCV NS5A protein expression. β-actin was used as loading control. Protein expression levels were quantified by densitometric analysis using Fiji software (Version 2.9.0) and expressed relative to the control condition. (**D**) Effect of Compound **2** in combination with the clinically approved HCV therapy Daclatasvir (DCV). The same cells were treated or not (Control) with Compound **2** (1 μM) in combination or not with DCV (6 pM). The effect of the two chemicals on viral propagation was determined, as described above. Statistical analysis was performed using one-way ANOVA followed by the appropriate post hoc analysis. **** *p* < 0.0001 vs. control.

**Figure 4:**
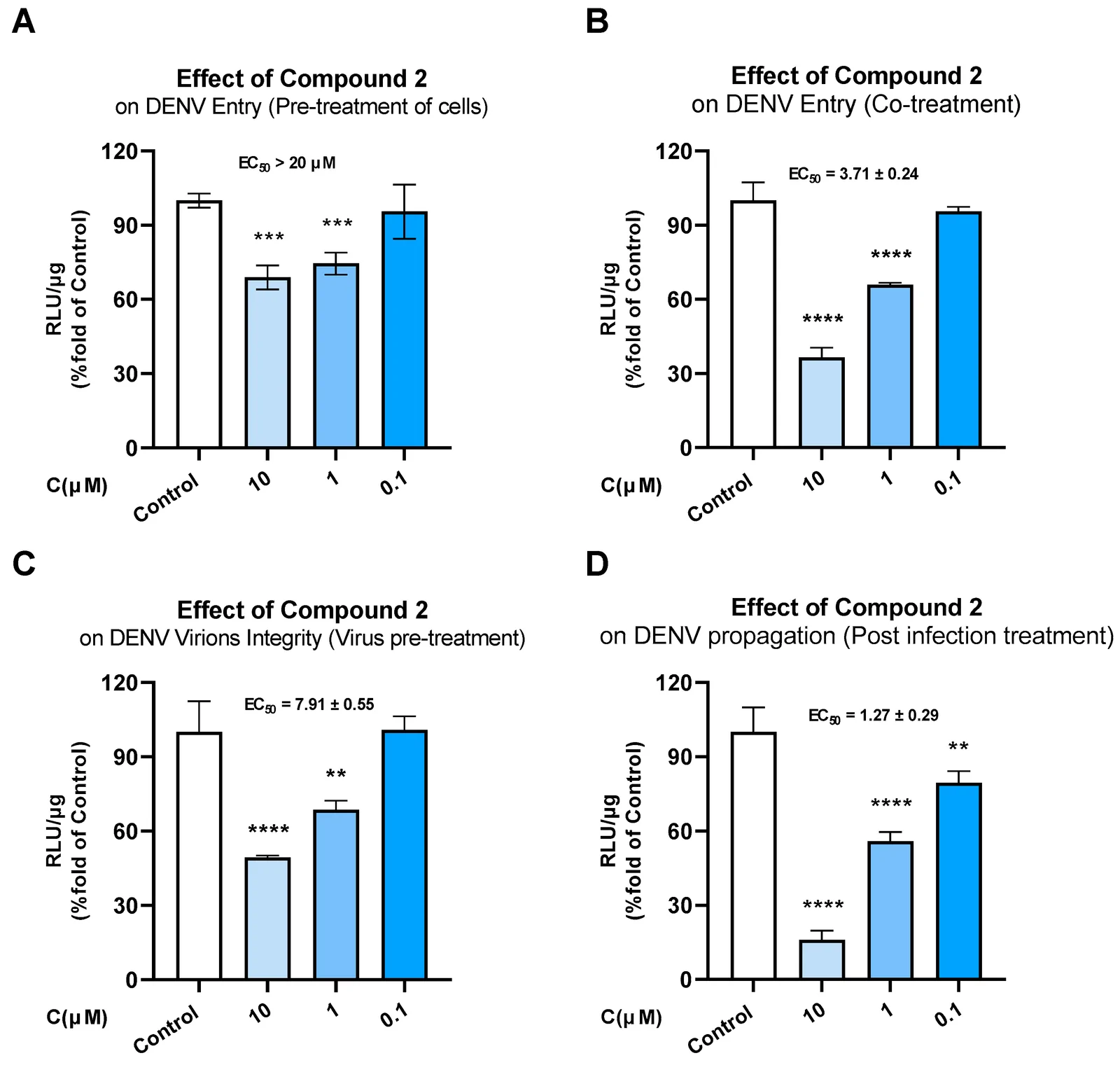
Effect of compound **2** on different stages of the DENV life cycle in Huh7.5 cells infected with DVR2A reporter virus. Cells or viral particles were exposed to serial dilutions of compound **2** under different treatment conditions to determine the stage of the viral life cycle affected by the compound. (**A**) Cell pre-treatment (entry assay): Huh7.5 cells were pre-treated with compound **2** for 2 h prior to infection, after which the compound was removed and cells were infected with DENV. (**B**) Co-treatment (entry assay): cells were infected with DENV in the continuous presence of compound **2** during the 4 h inoculation period. (**C**) Virion integrity assay: DENV-containing supernatants were pre-incubated with compound **2** for 2 h before infection of Huh7.5 cells. (**D**) Post-infection treatment (propagation assay): cells were infected with DENV for 4 h, after which the inoculum was removed and fresh medium containing compound **2** was added for the remainder of the experiment. Cells were harvested 48 h post-infection, and antiviral activity was determined by measuring firefly luciferase (F-Luc) activity normalized to total protein content. Results are expressed as percentage of DMSO-treated infected control cells. Statistical analysis was performed using one-way ANOVA followed by the appropriate post hoc analysis. ** *p* < 0.01, *** *p* < 0.001, **** *p* < 0.0001 vs. control.

**Figure 5:**
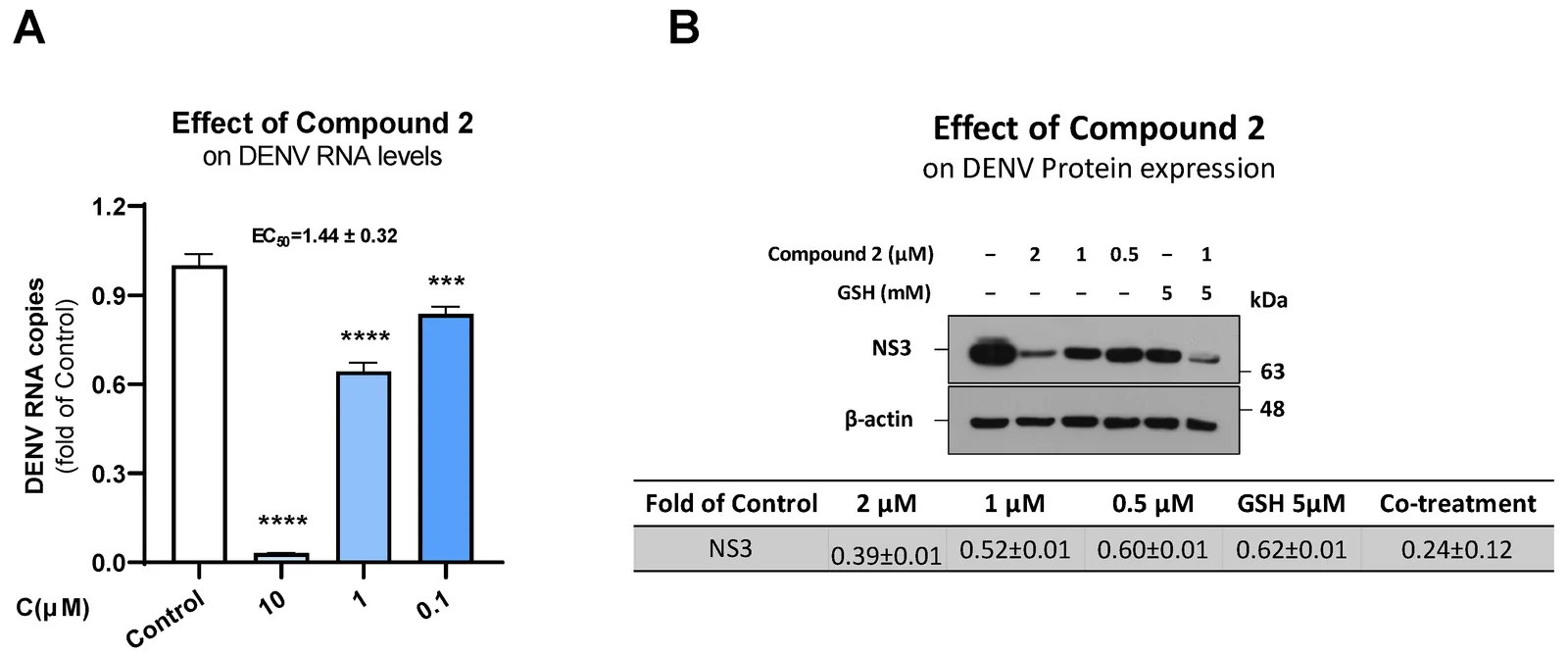
Effect of Compound **2** on DENV replication and its combination with reduced glutathione. Huh7.5 cells were infected with DVR2A (MOI = 0.05) for 4 h, and subsequently treated with the indicated concentrations of Compound **2,** reduced glutathione (GSH), or were mock-treated (Control). (**A**) Quantification of intracellular DENV RNA levels by RT-qPCR. Results are expressed relative to the Control cells and normalized to the mRNA levels of the endogenous control gene YWHAZ. Statistical analysis was performed using One-Way ANOVA followed by the appropriate post hoc analysis. *** *p* < 0.001, **** *p* < 0.0001 vs. control. (**B**) Western blot analysis was performed using the anti-NS3 monoclonal antibody. β-actin was used as loading control. Protein expression was quantified by densitometric analysis using Fiji software (Version 2.9.0) and expressed relative to the control condition. Results were obtained from three independent biological repetitions performed in triplicates. One representative blot is shown.

**Table 1:** ^1^H and ^13^C-NMR Spectral Data of Compounds **1** and **2**.

	Dehydrocostus Lactone (1)		Costunolide (2)	
	^1^H (Multiplicity, *J* in Hz)	^13^C	^1^H (Multiplicity, *J* in Hz)	^13^C
1	1.24–1.27 (1H, m); 1.39–1.47 (1H, m)	30.3	4.85 (1H, dd, *J* = 4.46, 11.36)	127.1
2	2.24–2.29 (1H, m)	36.3	2.20 (m); 2.25 (m)	26.2
3		149.3	2.02 (m); 2.24–2.32 (m)	39.5
4	—	52.0	—	140.1
5	2.93 (m)	85.3	4.74 (1H, d, *J* = 9.88)	127.3
6	3.96 (1H, t, *J* = 9.24)	47.6	4.56 (1H, dd, *J* = 8.84, 9.72)	81.9
7	1.87–1.96 (m)	31.0	2.59 (m)	50.4
8	2.49–2.53 (m), 2.86–2.95 (m)	32.5	1.64–1.70 (m); 2.05–2.11 (m)	28.1
9	2.14–2.29 (m)	139.8	2.45 (1H, dd, *J* = 13.56, 6.20)	41.0
10	—	45.1	—	137.0
11	—	151.3	—	141.5
12	—	170.3	—	170.5
13	5.51 (1H, d, *J* = 3.12), 6.23 (1H, d, *J* = 3.52)	120.2	5.54 (1H, d, *J* = 3.24); 6.27 (1H, d, *J* = 3.24)	119.7
14	4.92 (1H, brs), 4.83 (1H, brs)	109.6	1.44 (3H, s)	16.1
15	5.09 (1H, d, *J* = 1.59), 5.29 (1H, d, J = 1.89)	112.6	1.72 (3H, d, *J* = 1.44)	17.4

**Table 2:** Replicon assay results for compounds **1–2** against HCV and DENV.

Compound	CC_50_ ^b^ (μM), in Huh7 Cells	Huh5-2 (HCV Replicon)		Huh7-D2 (DENV Replicon)	
		EC_50_ ^a^ (μM)	SI ^c^	EC_50_ ^a^ (μM)	SI ^c^
Dehydrocostus lactone (**1**)	33.49 ± 0.17	2.34 ± 0.14	14.31	>10	-
Costunolide (**2**)	28.61 ± 0.15	2.06 ± 0.17	13.88	5.89 ± 0.22	4.86

^a^ EC_50_ values (half-maximal effective concentration) represent the mean ± SD of three independent experiments, and were calculated against HCV genotype 1b (Huh5-2, HCV 1b replicon cells) or DENV-2 (Huh7-D2, DENV-2 replicon cells), as indicated ^b^ CC_50_ values (half-maximal cytotoxic concentration against Huh5-2 cells, HCV replicon cells) represent the mean ± SD of three independent experiments. ^c^ Selectivity indices were derived from the ratio of the CC_50_ value to the EC_50_ value.

## Data Availability

The original contributions presented in this study are included in the article/Supplementary Materials. Further inquiries can be directed to the corresponding author(s).

## Supplementary Materials

The following supporting information can be downloaded at https://www.mdpi.com/article/10.3390/v18101056/s1, Figure S1: ^1^H-NMR analysis of **1** (400 MHz, CDC_l3_); Figure S2: ^13^C-NMR analysis of **1** (100 MHz, CDC_l3_); Figure S3: HRMS spectrum of **1**; Figure S4: ^1^H-NMR analysis of **2** (400 MHz, CDCl_3_); Figure S5: ^13^C-NMR analysis of **2** (100 MHz, CDC_l3_); Figure S6: HRMS spectrum of **2**.
