# Supplementary material for: Antiviral Sesquiterpene Lactones from Laurus nobilis L.: Inhibition of Hepatitis C Virus and Dengue Virus Replication
Source: Viruses. 2026 Sep 23;18(10):1056. doi: 10.3390/v18101056 (PMC13709689; doi:10.3390/v18101056)
Supplement: Supplementary file 1 [file viruses-18-01056-s001.zip › viruses-4542692-supplementary.pdf]

Supplementary material

Antiviral Sesquiterpene Lactones from *Laurus nobilis* L.: Inhibition of Hepatitis C Virus and Dengue Virus Replication

Yhiya Amen [1,2], Marwa Elsbaey [1], George Mpekoulis [3], Evangelia Christou [3], Helena Tsimpili [4], Niki Vassilaki [3], Kuniyoshi Shimizu [2,5,*], Grigoris Zoidis [4,*]

[1] Department of Pharmacognosy, Faculty of Pharmacy, Mansoura University, Mansoura 35516, Egypt
[2] Department of Agro-Environmental Sciences, Graduate School of Bioresource and Bioenvironmental Sciences, Kyushu University, Fukuoka 819-0395, Japan
[3] Molecular Virology Laboratory, Hellenic Pasteur Institute, 11521 Athens, Greece
[4] Division of Pharmaceutical Chemistry, Department of Pharmacy, School of Health Sciences, National and Kapodistrian University of Athens, Panepistimiopolis-Zografou, 15771 Athens, Greece
[5] Institute of Asian and Oceanic Studies, Kyushu University, Fukuoka, Japan
* Correspondence: shimizu.kuniyoshi.381@m.kyushu-u.ac.jp (K.S.); zoidis@pharm.uoa.gr (G.Z.)

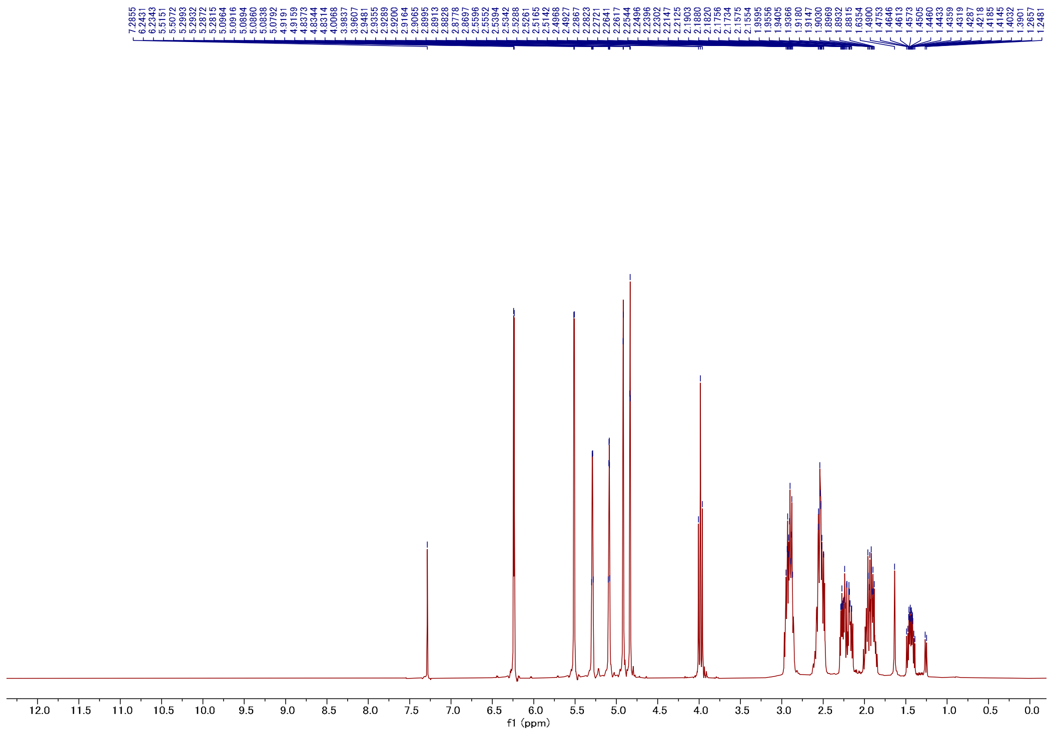

Figure S1: ^{1}H-NMR analysis of **1** (400 MHz, $CDCl_3$)

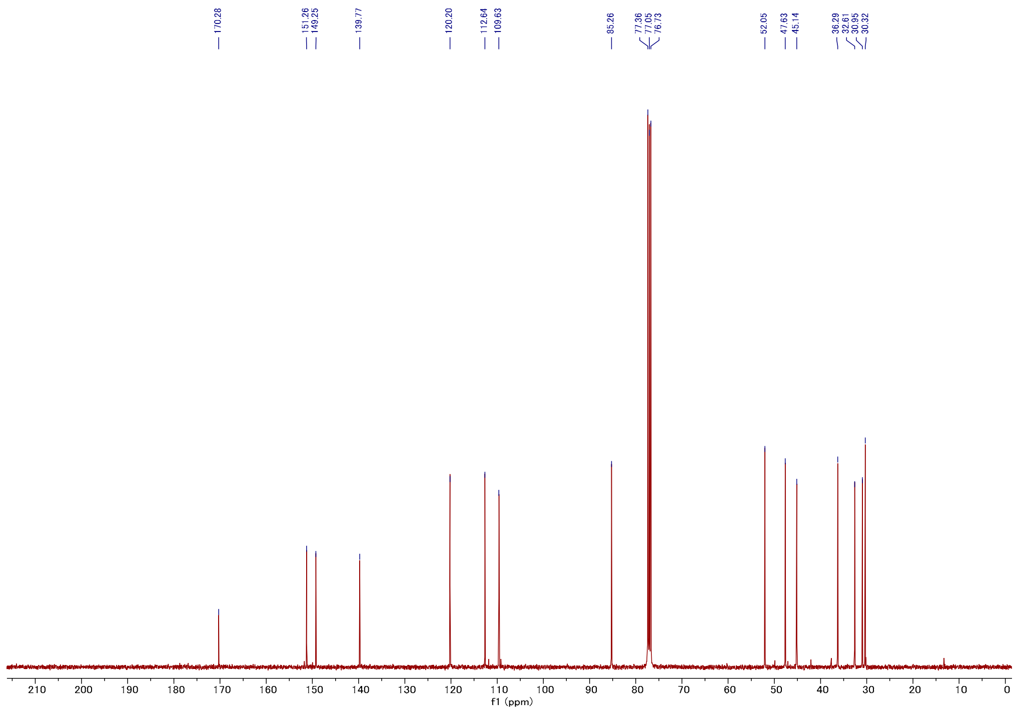

Figure S2: ^{13}C-NMR analysis of **1** (100 MHz, $CDCl_3$)

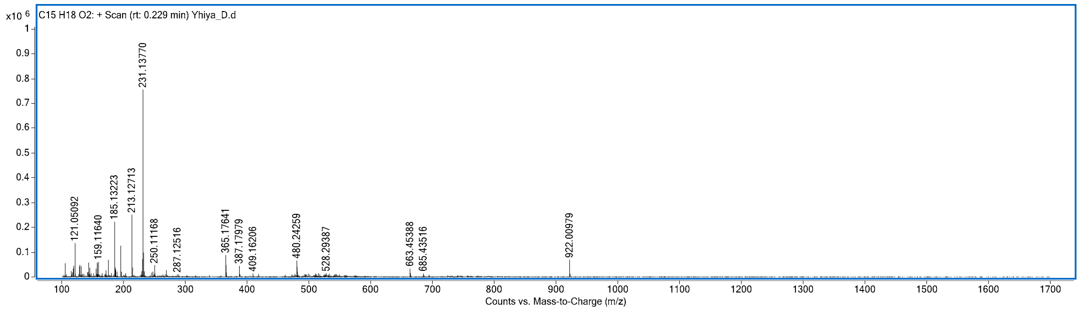

Figure S3: HRMS spectrum of **1**

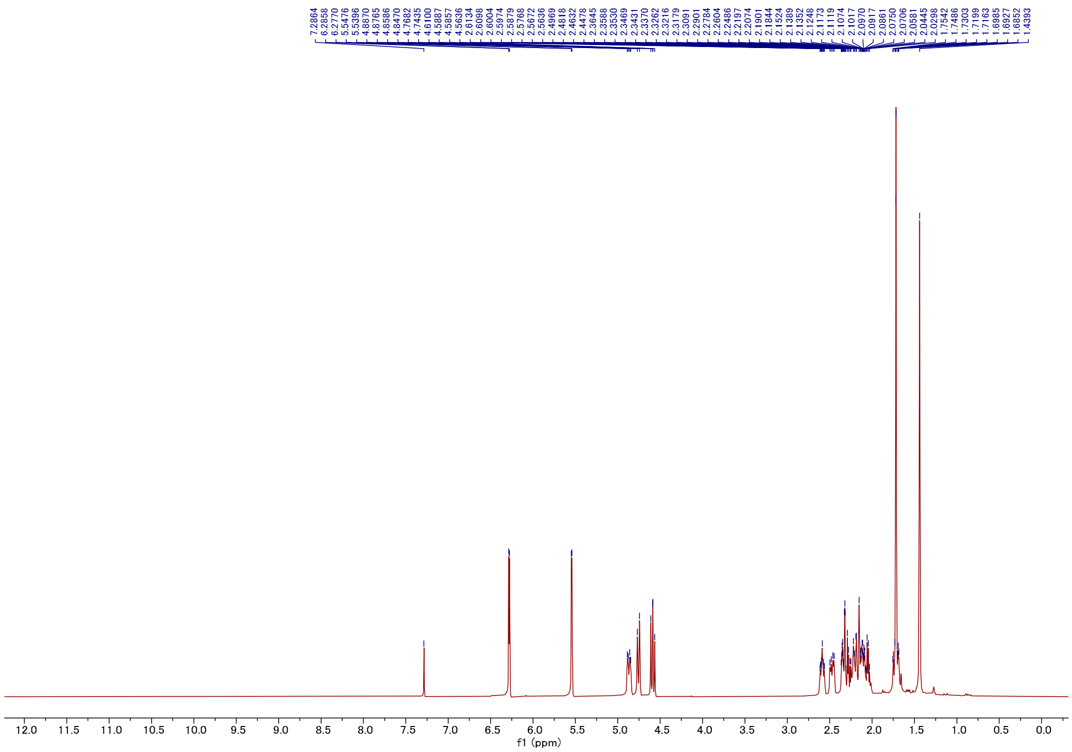

Figure S4: ^{1}H-NMR analysis of **2** (400 MHz, $CDCl_3$)

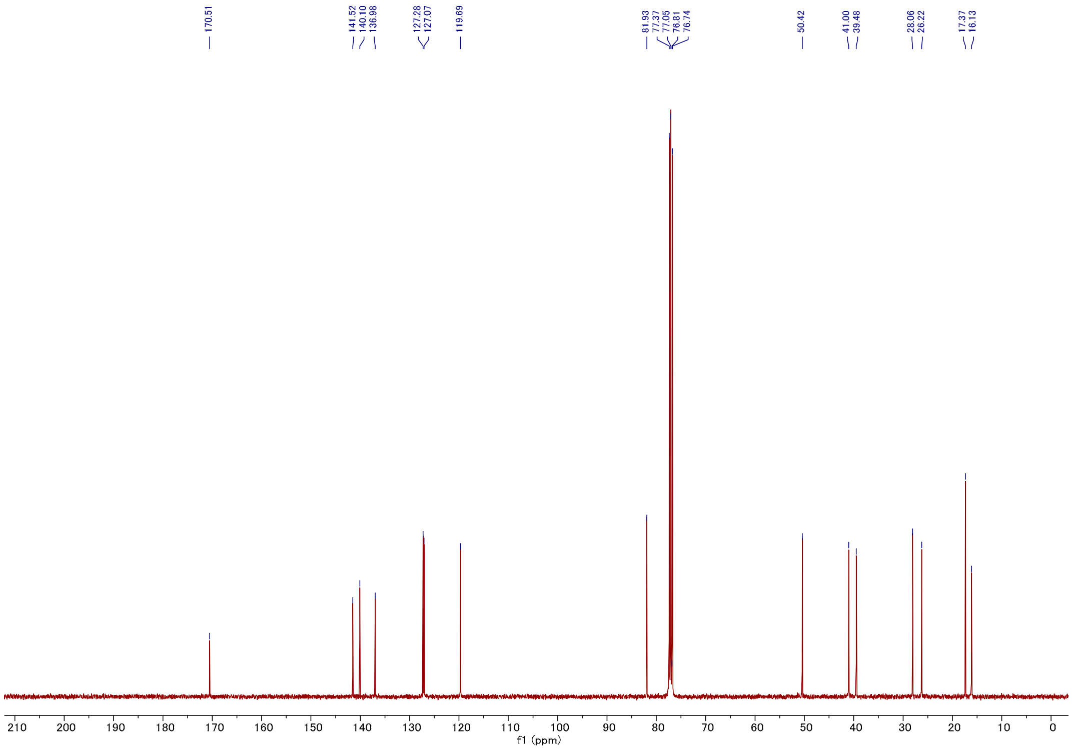

Figure S5: ^{13}C-NMR analysis of **2** (100 MHz, $CDCl_3$)

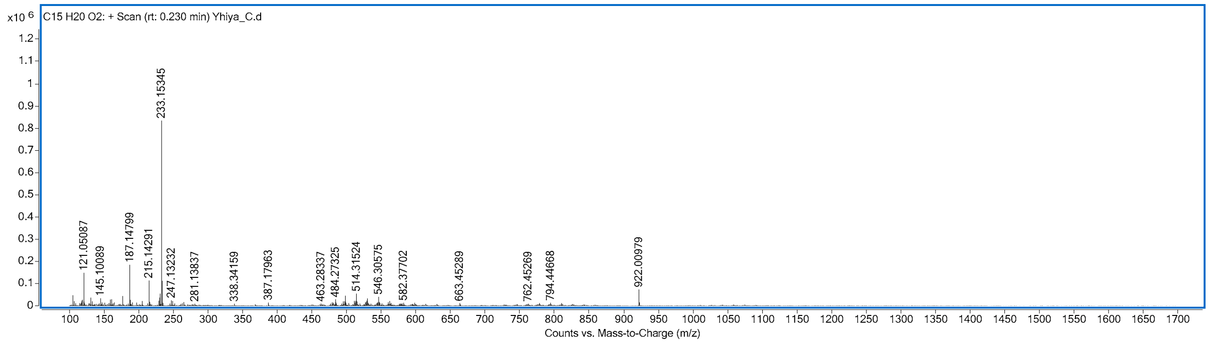

Figure S6: HRMS spectrum of **2**
